# Massive hemorrhage arising of inferior thyroid artery by radiofrequency ablation for secondary hyperparathyroidism: Two case reports

**DOI:** 10.1097/MD.0000000000031952

**Published:** 2022-12-09

**Authors:** Xiaoling Liu, Xiachuan Qin, Xiaomin Hu, Zhihua Wang

**Affiliations:** a Department of Ultrasound, Second Clinical Medical College of North Sichuan Medical College, Nanchong Central Hospital, Nanchong, China; b North Sichuan Medical College, Nanchong, China; c Chengdu Medical College, Chengdu, China.

**Keywords:** case report, inferior thyroid artery, pseudoaneurysm, radiofrequency ablation, secondary hyperparathyroidism

## Abstract

Secondary hyperparathyroidism (SHPT) is a common complication of chronic kidney disease. Radiofrequency ablation (RFA) is a safe and minimally invasive treatment for SHPT, which can effectively reduce the level of parathyroid hormone (PTH). Inferior thyroid artery (ITA) is a rare and dangerous complication, We report two cases of ITA bleeding caused by RFA. Intraoperative contrast-enhanced ultrasound (CEUS) can accurately show the source and scope of bleeding. Ultrasound guided RFA and compression hemostasis were successful.

**Interventions:**

During the operation, CEUS was used to detect ITA bleeding, bleeding range and location quickly and accurately at the early stage, and ultrasound guided compression and RFA were used to treat small bleeding points. ITA bleeding was timely and effectively controlled, and the bleeding range was limited to pseudoaneurysm.

**Outcomes:**

The patient received antiplatelet and anticoagulant therapy for 2 days, and the pseudoaneurysm was filled with thrombus 36 hours and 72 hours after surgery. Later, the ultrasonography examination showed that the hematoma was gradually absorbed and contracted.

**Conclusion:**

Although RFA is a safe and minimally invasive treatment for secondary hyperparathyroidism, rupture and bleeding of the ITA are rare and dangerous. CEUS can quickly and accurately judge bleeding, bleeding range and location in the early stage. Ultrasound guided compression and RFA of small ITA bleeding points can timely and effectively control bleeding, limit the bleeding range to pseudoaneurysms, and close themselves.

## 1. Introduction

In recent years, ultrasound-guided radiofrequency ablation (RFA) has achieved satisfactory results in the treatment of thyroid and parathyroid diseases^.[1–5]^ However, the complications included recurrent laryngeal nerve injury, and hemorrhage, and the result is controllable and recoverable in most cases. Damage to the inferior thyroid artery (ITA) is extremely rare, and the condition is dangerous and fatal. For this rare complication, more suggestions are surgical ligation and/or excision and transcatheter arterial embolization.^[6–9]^ However, whether it is surgery or intervention, has caused additional harm to the patient. Here, we introduce two cases of massive hemorrhage of the ITA caused by secondary hyperparathyroidism (SHPT) RFA, which can be effectively controlled by compression and RFA, avoiding surgery or intervention.

## 2. Case 1

A 53-year-old woman had a history of hypertension for more than 10 years and hemodialysis for 7 years. The parathyroid hormone level was >5000 pg/mL (normal range is 15–65 ng/mL). Her hip and lower limb pain had gradually developed a year ago. She was administered calcimimetics for treatment, but the curative effect was poor. Ultrasonography (US) indicated that the size of the upper and lower parathyroid glands in the left neck was about 16.5 mm × 7.7 mm, 14.2 mm × 8.6 mm, and that of the upper and lower parathyroid glands in the right neck was about 7.4 mm × 3.9 mm, 18.4 mm × 9.9 mm. The patient was then asked to undergo RFA.

The patient was dialyzed for 24 hours before ablation. RFA of the hyperplastic parathyroid glands was performed using a radiofrequency generator (Taishanglide, Mianyang, China) along with an 18-gauge monopolar internally cooled electrode (Taishanglide). Under ultrasound guidance, 1% lidocaine was injected for superficial nerve block anesthesia of the neck.

First, the two glands on the left side were ablated. After 10 minutes of rest, the upper right gland was ablated. When the needle was inserted in the right lower gland, the patient experienced sudden nausea and severe vomiting. RFA was immediately stopped.

**Figure 1.**
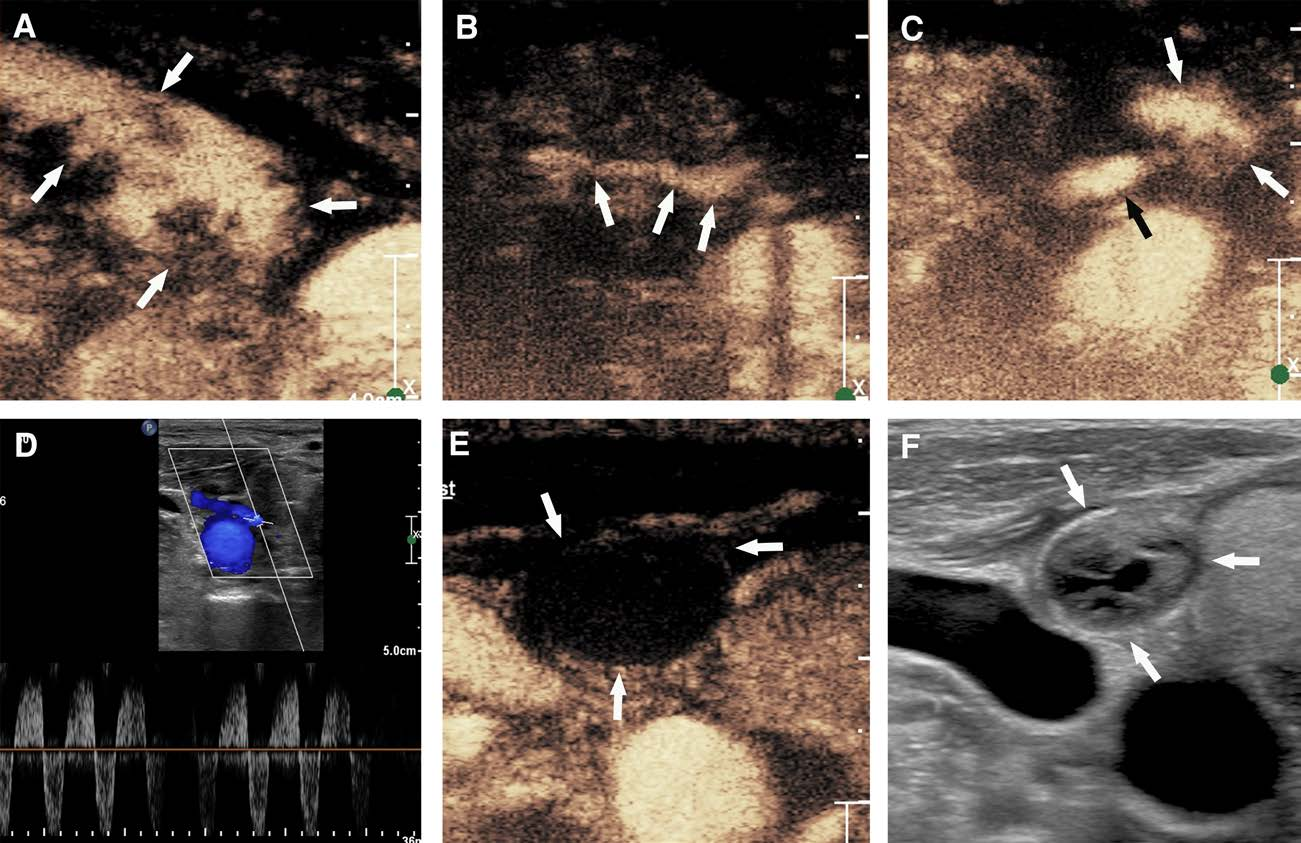
US Scans of the bleeding before, during, and after Radiofrequency Ablation. CEUS showed that Acute hemorrhage of right neck (Panel A, white arrow), the source of bleeding is the inferior thyroid artery (Panel B, arrow). After ablation, the enhancement range was significantly reduced, Pseudoaneurysm formation (Panel C, white arrow),the neck of the pseudoaneurysm (Panel C, black arrow).Spectral Doppler revealed a typical to-and-fro flow (bidirectional) (Panel D).Thirty-six hours later, CEUS showed that a thrombus filled the whole cavity (Panel E, white arrow). US showed that the hematoma was smaller (Panel F, white arrow). CEUS = contrast-enhanced ultrasound, US = ultrasonography.

After resting for approximately 3 minutes, the patient’s right neck was markedly enlarged, with mild dyspnea. The patient’s blood pressure was as high as 220/170 mm Hg, to provide antihypertensive treatment. Simultaneously US revealed a large hematoma in front of the right thyroid. Contrast-enhanced ultrasound (CEUS) showed active bleeding from the right ITA (Fig. 1, Panel A and B). We immediately performed a partial compression treatment on the right side for about 5 minutes. but it was not efficient, so we decided to use RFA to stop bleeding.

Under CEUS guidance, the ablation needle was inserted at the bottom of the bleeding point for ablation (60 W). After cauterization, CEUS indicated that the bleeding had stopped, but the formation of a pseudoaneurysm was 18 × 11 × 29 mm^3^ in size (Fig. 1, Panel C). Since the pseudoaneurysm is located on the lateral side of the thyroid and originates from the inferior thyroid artery, we are worried that further ablation will destroy the tumor wall and cause rebleeding. The pseudoaneurysm in the inferior thyroid artery remained unchanged after 1 hour of dynamic US. Color Doppler imaging (CDFI) showed rotational blood flow in the anechoic part, and spectral Doppler analysis showed typical round-trip blood flow. The blood flow velocity was 80 cm/s (Fig. 1, Panel D). The patient received antiplatelet and anticoagulant therapy for 2 days.

Because the patient refused thrombin injection or coil embolization, we chose to perform ultrasound examination every 12 hours, and closely monitored their blood pressure and breathing. Fortunately, the pseudoaneurysm whose size was 14.7 × 9.4 × 25 mm^3^ was filled with thrombus at 36 hours after the procedure (Fig. 1, Panel E and F). A week later, US examination showed that the hematoma had been slightly absorbed and contracted.

## 3. Case 2

A 46-year-old man had a history of hypertension of 9 years. The parathyroid hormone level was 2246 pg/mL. Because the patient’s heart was enlarged and left ventricular systolic function was reduced (EF = 44%), the patient was asked to undergo RFA.

The patient was dialyzed for 24 hours before RFA. First, the gland on the left side was ablated. Second, the upper right gland was RFA. During the entire RFA process, a total of 100 mL of normal saline was injected as an isolation belt to protect the surrounding nerves and blood vessels, and we found that the left neck was significantly larger than the right and harder.

**Figure 2.**
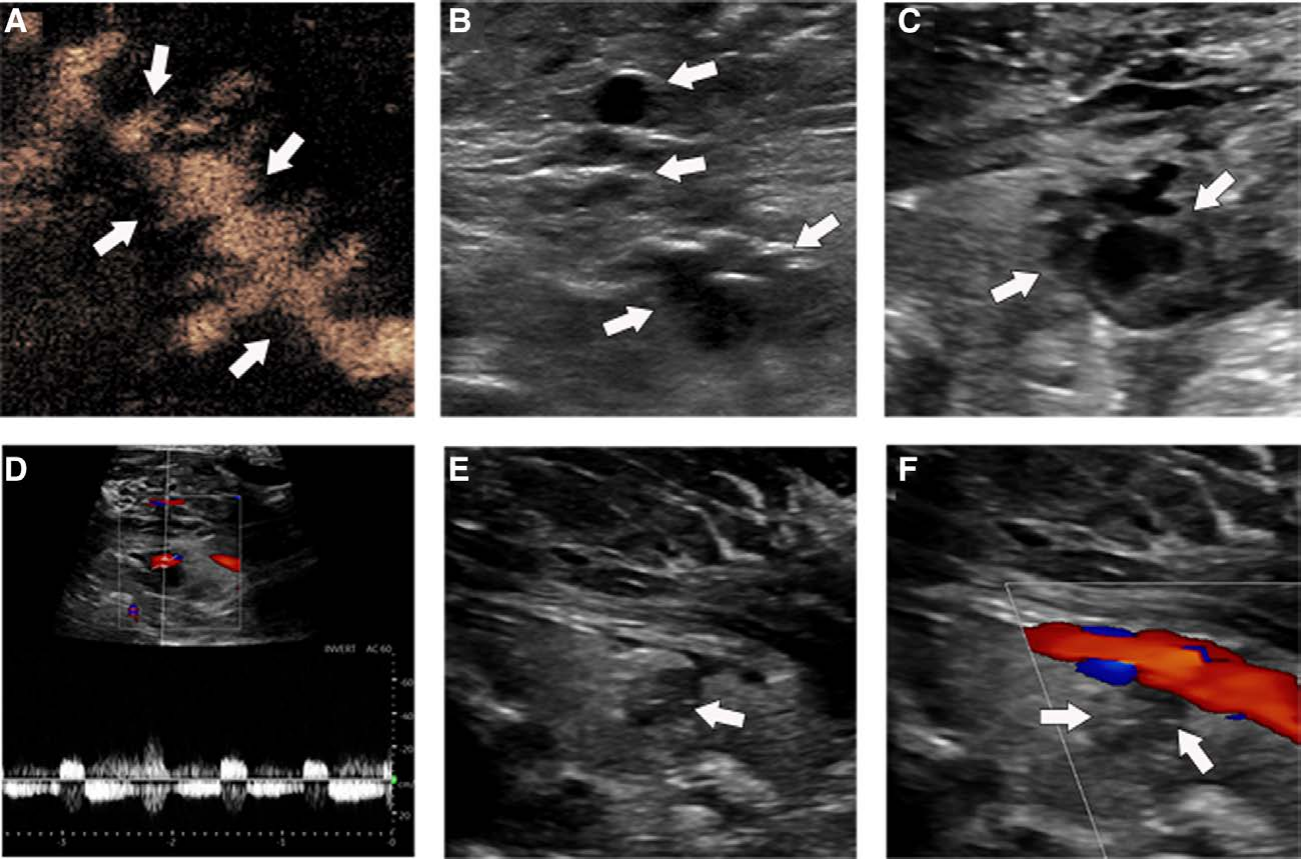
CEUS showed that Acute hemorrhage of left neck, the source of bleeding is the inferior thyroid artery (Panel A, white arrow). We can see more anechoic areas (Panel B, white arrow) on the outside of the thyroid gland, 24 hours later, a pseudoaneurysm from the inferior thyroid artery, with a size of, and thrombosis on the capsule wall (Panel C). Spectral Doppler revealed a typical to-and-fro flow (bidirectional) (Panel D). 72 hours later, US showed that a thrombus filled the whole cavity, and smaller than before (Panel E, white arrow) CDFI showed that no blood flow entered the tumor (Panel F, white arrow). CDFI = Color Doppler imaging, CEUS = contrast-enhanced ultrasound, US = ultrasonography.

Simultaneously, US revealed a large hematoma in front of the left thyroid. CDFI revealed abnormal blood flow signals in the ITA. Furthermore, CEUS showed active bleeding from the left ITA (Fig. 2, Panel A). Us found that there was no echo in many places outside the left thyroid gland (Fig. 2, Panel B). Then we stopped the bleeding point around the breach of ITA by RFA (60 W), and located the bleeding point according to US, Immediately we took a partial compression treatment on the left about 20 minutes.

After ultrasound, we found that many previous bleeding points had closed. Although there was still active bleeding downward from the ITA, the scope was limited, with a pseudoaneurysm size of approximately 12 × 10 × 12 mm^3^ was formed. Spectral Doppler analysis showed a typical round-trip blood flow. Blood flow velocity was 50 cm/s. At the same time, the neck hardness of the patient was significantly softer than before, there was no dyspnea, and the heart rate and blood oxygen levels were stable. We chose to closely watch. The patient received antiplatelet and anticoagulant therapy for 2 days.

After 24 hours, US reexamination showed that the size of the pseudoaneurysm was approximately 30 × 22 × 30 mm^3^ with a 19 × 12 × 15 mm^3^ thrombus in the wall. At the same time, the blood flow velocity was 30 cm/s, which was significantly lower than that on the previous day (Fig. 2, Panel C and D). After 72 hours, the pseudoaneurysm with a size of 15 × 9 × 13 mm^3^ was filled with thrombus and no blood flow (Fig. 2, Panel E and F).

## 4. Discussion

SHPT is ubiquitous in patients with chronic renal failure treated by with long-term dialysis.^[10]^ It has been reported that ablation of SHPT has achieved good results,^[4,11–14]^ and under ultrasound guidance, the liquid isolation zone was established, and the hyperplastic parathyroid tissue was ablated. Although minimally invasive, it is associated with a greater risk than thyroid RFA. Attention should be paid to sudden swelling of the neck during surgery. CEUS can be used to quickly and sensitively diagnose active ITA bleeding. According to the instructions for the contrast medium, we could accurately locate the ITA bleeding point.

We then analyzed the cause of ITA bleeding in the patient. Intraoperative cough may have caused the puncture needle to puncture the ITA, because the parathyroid gland is often near the ITA. Both patients were uremic. Long-term dialysis and parathyroid hyperplasia lead to more brittle blood vessels than normal blood vessels. At the same time, most patients had abnormal hemagglutination, even if the examination report showed that they were normal. Third, the establishment of an intraoperative liquid isolation zone reduces pressure on the ITA. Once damaged, bleeding may become more severe because there is no pressure from the surrounding tissue. Mild subcutaneous edema and recurrent nerve injury are common complications, but most patients can be recovered.^[4]^ Acute active bleeding is often dangerous. The hematoma in the neck, which can potentially compress the airway^[8,15]^ and even cause death,^[16,17]^ especially in the case of severe respiratory distress, airway management alone is insufficient, and emergency surgery is needed.^[18]^

However, our assessment of patient bleeding was rapid and accurate. Owing to the limited clearance space of the posterior thyroid tissue, the pressure increases with the increase in bleeding, which may in turn compress the bleeding vessels. At the same time, under the guidance of ultrasound, we provided the patient with sufficient pressure on the bleeding side neck for a long time, because it is a cost-effective way to treat ITA bleeding, which can be carried out in any ward and at any time.^[19]^ Because bleeding was caused during ablation, we performed radiofrequency ablation around the bleeding point at the same time, because RFA waves act to increase the contractile force of collagen, which may cause tissue structure closure, and can also promote thrombosis.^[14]^ Although there have been reports of attempts to treat thyroid pseudoaneurysms with RFA,^[20]^ the tumor was small and in the thyroid. The ITA is thick, and there is no surrounding tissue. We believe that in this case, it is safer to ablate the small bleeding points, we dare not continue to try, fearing that ablation may rupture aneurysms and cause greater and more violent bleeding.

In the current experience, it is considered that the active coil embolization or surgical treatment of the rupture of the ITA. However, we still believe that they should not be a first-line approach, and that, the flow velocity of the inferior thyroid artery is lower than that of other peripheral blood vessels.

## 5. Conclusion

Although RFA is a safe and minimally invasive treatment for secondary hyperparathyroidism, rupture and bleeding of the ITA are rare and dangerous. CEUS can quickly and accurately judge bleeding, bleeding range and location in the early stage. Ultrasound guided compression and RFA of small ITA bleeding points can timely and effectively control bleeding, limit the bleeding range to pseudoaneurysms, and close themselves.

## Acknowledgments

The authors declare that they have no relationships with any company.

## Author contributions

**Data curation:** Xiaomin Hu, Zhihua Wang.

**Formal analysis:** Xiaoling Liu.

**Investigation:** Xiaomin Hu, Zhihua Wang.

**Methodology:** Xiachuan Qin.

**Project administration:** Xiaoling Liu, Xiachuan Qin.

**Supervision:** Xiachuan Qin.

**Validation:** Xiaomin Hu, Zhihua Wang.

**Writing – original draft:** Xiaoling Liu.

**Writing – review & editing:** Xiaoling Liu, Xiachuan Qin.
